# Overexpression of CIP2A is associated with poor prognosis in multiple myeloma

**DOI:** 10.1038/sigtrans.2017.13

**Published:** 2017-05-26

**Authors:** Xuewen Liu, Wei Cao, Shanshan Qin, Te Zhang, Junnian Zheng, Ying Dong, Pinghong Ming, Qian Cheng, Zheng Lu, Yang Guo, Baofu Zhang, Ying Liu

**Affiliations:** 1 Laboratory of Molecular Target Therapy of Cancer, Institute of Basic Medical Sciences; Hubei University of Medicine, Shiyan, China; 2 MOE Key Laboratory of Industrial Fermentation Microbiology, College of Biotechnology, Tianjin University of Science and Technology, Tianjin, China; 3 Jiangsu Center for the Collaboration and Innovation of Cancer Biotherapy, Cancer Institute, Xuzhou Medical University, Xuzhou, China; 4 Department of Oncology, The Second Affiliated Hospital, College of Medicine, Zhejiang University, Hangzhou, China; 5 Department of Pathology, Zhuhai Hospital Affiliated with Jinan University, Zhuhai, China

## Abstract

Cancerous inhibitor of protein phosphatase 2A (CIP2A), an endogenous protein phosphatase 2A (PP2A) inhibitor, has been identified as an oncoprotein in promoting cancer initiation and progression of several types of cancer. However, the expression and the role played by CIP2A in the pathogenesis of multiple myeloma (MM) remain unclear. In this study, we showed that CIP2A was overexpressed in human MM cell lines and MM patients’ bone marrow tissues. Clinicopathologic analysis showed that CIP2A expression was significantly correlated with clinical stage and percent of plasma cells in bone marrow. Kaplan–Meier analysis revealed that patients with high CIP2A expression presented with poorer overall survival rates than those with low CIP2A expression. Moreover, CIP2A knockdown in MM cells resulted in attenuated proliferative abilities. In addition, CIP2A depletion sensitizes dexamethasone (Dex)-resistant cells to Dex. The effect of CIP2A on proliferation and Dex therapy was mediated by the inhibition of PP2A, which in turn activated Akt. *In vivo* studies confirmed that CIP2A regulated MM tumorigenesis and the phosphorylation of Akt. Taken together, our results suggest that CIP2A oncoprotein plays an important role in MM progression and could serve as a prognosis marker and a novel therapeutic target for the treatment of patients with MM.

## Introduction

As a neoplastic disorder of plasma cells characterized by clonal proliferation within the bone marrow (BM), multiple myeloma (MM) accounts for ~10% of all hematological cancers and ~1% of all cancer deaths.^1^ In 2017, the annual incidence of MM increased with the aging of the population worldwide. An estimated 30 280 new cases (17 490 in men and 12 790 in women) of MM were diagnosed in 2017, and 12 590 deaths (6660 in men and 5930 in women) are estimated to occur from this disease in the United States.^2^ Overall survival has improved in recent years. Patients are now predicted to have a median survival of ~5 years.^3^ Unfortunately, despite the improved therapeutic armamentarium, nearly all patients experience relapse and refractory disease. To date, MM remains an incurable disease. Therefore, there is an urgent need to explore novel therapeutic target and agent to improve the survival of patients with MM.

The oncogenic transformation of human cells requires the perturbation of a distinct set of oncogenes and tumor suppressors. Protein phosphatase 2A (PP2A) complexes function as tumor suppressors by inhibiting the activity of several critical oncogenic signaling pathways such as Akt and ERK.^4^ Consequently, inhibition of PP2A phosphatase activity is one of many prerequisites for the transformation of normal human cells into cancerous cells. However, the mechanisms underlying PP2A inactivation in human cancers are poorly understood. Cancerous inhibitor of protein phosphatase 2A (CIP2A), a recently identified endogenous PP2A inhibitor in malignant cells, is one possible mechanism. CIP2A stabilizes c-Myc protein by inhibiting its degradation mediated by PP2A in cancer cells.^5^ In addition to inhibiting c-Myc degradation, CIP2A is regulated in a positive feedback loop with c-Myc by promoting each other’s expression.^6^ Previous independent studies showed that aberrant overexpression of CIP2A is associated with tumor growth, resistance to apoptosis, drug resistance, prognosis and metastasis in many human solid malignancies including head and neck,^5^ gastric,^7^ breast,^8^ tongue,^9^ ovarian,^10^ and lung cancers.^11^ Besides, CIP2A downregulation and inactivation of the Akt pathway have been reported to inhibit the proliferation and to induce apoptosis in a variety of hematologic neoplasms.^12–14^ Importantly, recent studies provided evidence that high CIP2A expression is a poor prognostic factor in normal karyotype acute myeloid leukemia (AML).^12^ Barragán *et al.*^12^ showed that high CIP2A expression is a recurrent event in AML, where it represents a marker of reduced overall survival and a poor prognostic factor, as previously reported in other tumors. In addition, CIP2A depletion downregulates cell proliferation. Thus, CIP2A represents a novel therapeutic target in AML. Furthermore, CIP2A is associated with radioresistance and modulates the sensitivity of breast cancer cells to bortezomib treatments.^15^ Choi *et al.*^16^ determined that CIP2A expression is associated with the sensitivity to doxorubicin and CIP2A overexpression in MCF-7 cells overcame the inhibition of cell proliferation in response to doxorubicin treatment. Moreover, CIP2A knockdown may sensitize metastatic castration-resistant prostate cancer cells to cabazitaxel chemotherapy.^17^ Liu and colleagues^18^ also showed that CIP2A knockdown increased the drug sensitivity of HeLa and doxorubicin-resistant HeLa cells. In addition, CIP2A is involved in regulating multidrug resistance of cervical adenocarcinoma upon chemotherapy by enhancing P-glycoprotein expression through E2F1. Conclusively, De and colleagues^1^ reviewed that CIP2A acts as an ‘oncogenic nexus’ contributing to cancer development, cancer evolution and drug resistance, suggesting that CIP2A can be targeted in cancer. However, the role of CIP2A in MM tumorigenesis and metastasis remains less elusive. Recently, Yang *et al.*^19^ determined that CIP2A silencing inhibited proliferation and promoted apoptosis in MM cell lines RPMI 8226 and NCI-H929 cells.

In this study, we analyzed the expression of CIP2A in MM specimens and cell lines by real-time quantitative PCR, western blot and immunohistochemistry. We also measured the effect of CIP2A on MM cell proliferation and dexamethasone (Dex)-mediated inhibition of cell proliferation in Dex-resistant cell lines. Our findings uncover the mechanisms of CIP2A-mediated tumorigenesis and Dex therapy.

## Materials and methods

### Patients

MM patient’s samples were obtained from the Department of Oncology, the Second Affiliated Hospital, College of Medicine, Zhejiang University (Hangzhou, China); Department of Pathology, Zhuhai Hospital Affiliated to Jinan University (Zhuhai, China); and Department of Hematology, Nanfang Hospital Affiliated to Nanfang Medical University (Guangzhou, China) after informed consent. Forty-one patients were studied. The diagnosis was based on clinical data and examination of BM according to French-American-British classification. CD138^+^ cells from 11 MM patients were isolated with informed consent from BM mononuclear cells using positive immunomagnetic column separation (Miltenyi Biotech, Auburn, CA, USA). The purity of CD138+ cells is above 97% as determined by flow cytometry. Peripheral blood mononuclear cells from four healthy donors were separated by Ficoll-Hipaque density sedimentation.^20^ Real-time quantitative PCR was used to detect these 11 MM patients and 4 healthy donors. Forty-one MM patients’ BM and 22 adjacent normal bone trabecula and BM paraffin section were detected by immunohistochemical analysis.

Immunohistochemical analysis as well as the scoring of immunoreactivity was performed using the mouse polyclonal anti-CIP2A antibody. The intensity of CIP2A staining was scored as 0 (no signal), 1 (weak), 2 (moderate) and 3 (marked). Percentage scores were assigned as 1, 10–30%; 2, 30–60%; and 3, 60–100%. The scores of each tumor sample were multiplied to give a final score of 1–3, and the tumors were finally determined as negative (−), score=0; lower expression (+), score=1; moderate expression (++), score=2; and high expression (+++), score=3. Tumor sample scored (++) to (+++) were considered positive (overexpression).

### Cells and reagents

The human MM cell lines U266 and RPMI 8226 (8226), and the pro-myelocytic cell line HL-60 were purchased from American Type Culture Collection (Manassas, VA, USA). Dex-sensitive (MM.1S) and Dex-resistant (MM.1R) human MM cell lines were kindly provided by Professor Jie Jin (Department of Hematology, The First Affiliated Hospital, Zhejiang University College of Medicine). All cell lines were incubated in a humidified CO_2_ incubator (5% CO_2_, 37 °C).

### Real-time quantitative PCR

Expression of the *CIP2A* gene was examined by real-time quantitative PCR (QPCR) normalized to the expression of GAPDH. Total RNA was extracted from cell lines or patients’ cells using the Trizol reagent (Invitrogen, Carlsbad, CA, USA). QPCR was performed using SYBR Premix Ex Taq (Perfect Real Time; TaKaRa Biotechnology, Shiga, Japan), according to the manufacturer’s instruction.^21^ For QPCR, we used *CIP2A* gene forward primer 5′- TGCGGCACTTGGAGGTAATTTC-3′, *CIP2A* gene reverse primer 5′- AGCTCTACAAGGCAACTCAAGC-3′; *GAPDH* forward primer 5′- TGTTGCCATCAATGACCCCTT-3′, *GAPDH* reverse primer 5′- CTCCACGACGTACTCAGCG-3′.

### Western blot

Cell pellets were lysed in radioimmunoprecipitation assay buffer containing 50 mM Tris (pH 8.0), 150 mM NaCl, 0.1% SDS, 0.5% deoxycholate, 1% NP-40, 1 mM dithiothreitol, 1 mM NaF, 1 mM sodium vanadate, 1 mM PMSF (Sigma-Aldrich, St Louis, MO, USA) and 1% protease inhibitors cocktail (Merck, Millipore, Kenilworth, NJ, USA). Protein extracts were quantitated and loaded on 8–12% SDS-polyacrylamide gel, electrophoresed and transferred to a polyvinylidene difluoride membrane (Millipore, Kenilworth, NJ, USA). The membrane was incubated with primary antibody, washed and incubated with horseradish peroxidase-conjugated secondary antibody (Pierce Biotechnology Inc., Rockford, IL, USA). Detection was performed using a chemiluminescent western detection kit (Cell Signaling Technology, Danvers, MA, USA). The antibodies used were anti-CIP2A, anti-phospho-Akt (Ser473), anti-Akt (Santa Cruz Biotechnology, Santa Cruz, CA, USA), anti-PP2Ac, anti-caspase-3, anti-caspase-9, anti-PARP (Cell Signaling Technology), anti-c-Myc (Proteintech Group, Inc, Rosemont, IL, USA) and anti-GAPDH (Abmart, Shanghai, China) antibodies.

### Transfection of DNA, siRNA and stable cell line generation

The pOTENT-1-CIP2A expression plasmid was purchased from Youbio Co. (Changsha, China). Transfection of the pOTENT-1-CIP2A plasmid into MM cells were carried out using Lipofectamine 3000 transfection reagent (Invitrogen) following the manufacturer’s protocol.

Two short interfering RNAs (siRNAs) targeting CIP2A were designed and synthesized by Shanghai GenePharma Co., referred to as siRNA1 and siRNA2. The siRNA sequences were as follows: 5′- CUGUGGUUGUGUUUGCACUTT-3′ (CIP2A siRNA1), 5′- ACCAUUGAUAUCCUUAGAATT-3′ (CIP2A siRNA2), 5′- UUCUCCGAACGUGUCACGUTT-3′ (negative control siRNA). Using the HiPerFect Transfection Reagent (Qiagen, Germantown, MD, USA), according to the manufacturer’s instructions, MM cells were transfected with 100 nM siRNA. And, 48 h after transfection, the cells were then collected for western blot, cell viability and CCK-8 assay.^22^ The CIP2A knockdown U266 cell line (CIP2A^KD^-U266) was established using Lipofectamine 3000 transfecting with CIP2A shRNA expression vector pGPU6/GFP/Neo-CIP2A (GenePharma, Shanghai, China). The sequences were as follows: negative control short hairpin RNA, 5′- GTTCTCCGAACGTGTCACGT-3′; shCIP2A 1#, 5′- TGCGGCACTTGGAGGTAATTT-3′; and shCIP2A 2#, 5′- GACAGAAACTCACACGACTAT-3′.

### RNA preparation and reverse transcription PCR

The total RNA of the cells was isolated using the TRIZOL Reagent (Invitrogen) and the phenol–chloroform extraction method, according to the manufacturer’s instruction. Total RNA (2 μg) was annealed with random primers at 65 °C for 5 min. The complementary DNA was synthesized using a 1st-STRAND cDNA Synthesis Kit (Fermentas, Hanover, MD, USA). For PCR amplification, primers are as follows: GAPDH, forward primer: 5′- TCACCAGGGCTGCTTTTA-3′, reverse primer: 5′- AAGGTCATCCCTGAGCTGAA-3′; CIP2A, forward primer: 5′- CCATATGCTCACTCAGATGATGT-3′, reverse primer: 5′- GTGTATCATCTCCACAGAGAGTT-3′.

### Cytotoxic assay and cell viability

Cells were seeded into a 96-well plate and precultured for 24 h, and then transfected with plasmid or siRNA for 24 or 48 h. Cell cytotoxicity was determined by 3-(4,5-dimethyl-2-thiazolyl)-2,5-diphenyl-2-H-tetrazolium bromide (MTT) or CCK-8 assay. Cell viability was estimated by trypan blue dye exclusion.^18^

### PP2A phosphatase activity

PP2A immunoprecipitation phosphatase assay kit (Upstate, Temecula, CA, USA) was used to measure phosphate release as an index of phosphatase activity, according to the manufacturer’s instructions.^23^

### Flow cytometric assays for Annexin-V

Cell apoptosis was evaluated by the Annexin-V detection using an AV-FITC kit (BD Biosciences, San Jose, CA, USA), according to the manufacturer’s instructions.^20^

### Murine model

All mice used in this study were bred and maintained in a specific pathogen-free environment. BALB/c nude mice (6–7 weeks old) were purchased from Hunan SJA Laboratory Animal Co., Ltd (Changsha, China). All animal studies were conducted according to the protocols approved by the Hubei University of Medicine Animal Care and Use Committee, complying with the rules of Regulations for the Administration of Affairs Concerning Experimental Animals (Approved by the State Council of China). The mice were injected subcutaneously with 1×10^7^ U266 (*n*=7), CIP2A^KD^-U266 (*n*=8), 8226 (*n*=7) or CIP2A^OE^-8226 (*n*=8) cells in 100 μl RPMI-1640 media into the right flank. Caliper measurements of the longest perpendicular tumor diameters were performed twice a week to estimate the tumor volume, using the following formula: 4*π*/3× (width/2)^2^× (length/2), representing the three-dimensional volume of an ellipse. Animals were killed when tumors reached 1.5 cm or if the mice appeared moribund to prevent unnecessary morbidity to the mice. At the time of the animals’ death, tumors were excised; cells were separated and subjected to PP2A activity analysis or lysed for western blot.

### Statistical analysis

All experiments were repeated at least three times and the data were presented as the mean±s.d. unless noted otherwise. Differences between data groups were evaluated for significance using the Student’s *t*-test of unpaired data or one-way analysis of variance and Bonferroni post test. For patients’ tissue samples, statistical analysis was performed with SPSS 20 software (SPSS, Inc, Chicago, IL, USA). The association between CIP2A staining and the clinicopathologic parameters of the MM patients, including age, gender, type of myeloma, BM plasma cells, platelet, hemoglobin, albumin, β_2_ microglobulin and International Staging System stage, was evaluated by the *χ*^2^ test. Differences were considered significant when *P*<0.05.

## Results

### CIP2A is overexpressed in MM and is associated with poor prognosis

**Figure 1:**
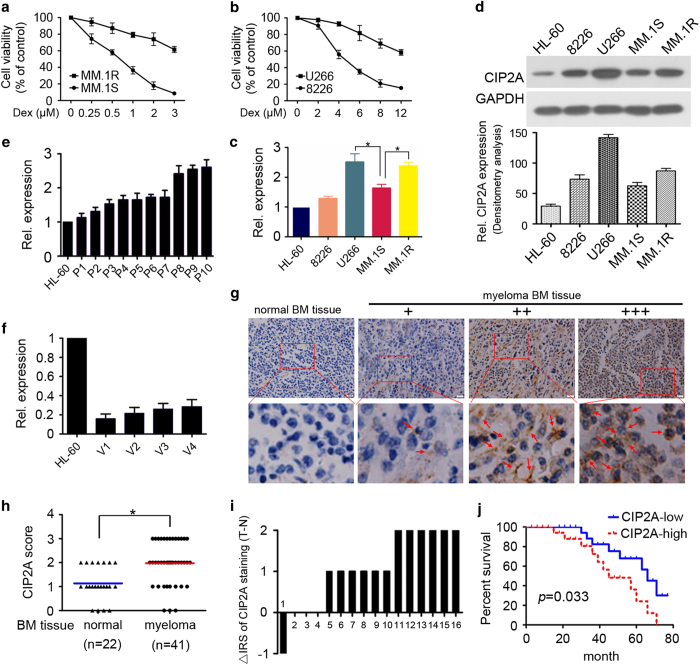
CIP2A is overexpressed in MM and is associated with poor prognosis. (**a**) Cell viability was determined by CCK-8 assay after Dex treatment in MM.1S and MM.1R cells for 24 h. (**b**) Cell viability was determined by CCK-8 assay after Dex treatment in 8226 and U266 cells for 24 h. (**c**) *CIP2A* relative expression determined by QPCR (compared to GAPDH, respectively). *P<0.05. (**d**) Western blot analysis showing CIP2A expression in different cell lines. The pro-myelocytic cell line HL-60 was used as a positive control. (**e**) *CIP2A* relative expression in patient samples (P1–10) determined by QPCR (compared to GAPDH, respectively). HL-60 was used as a positive control. (**f**) CIP2A relative expression in volunteers (V1–4), determined by QPCR (compared to GAPDH, respectively). HL-60 was used as a positive control. (**g**) Schematic images of immunohistochemistry staining intensities for CIP2A expression in MM and normal BM tissue. (**h**) Scores of immunohistochemistry staining for CIP2A expression in different stages of MM. *P<0.05. (**i**) The distribution of the difference in CIP2A staining (ΔIRS=IRST−IRSN). Immunoreactivity score (IRS) of CIP2A staining was available from 16 pairs of tissues; *P*-values were calculated with the Wilcoxon test. CIP2A expression was higher in tumor tissues (T) compared with paired adjacent non-tumor tissues (N). IRST, IRS of tumor tissues; IRSN, IRS of non-tumor tissues. *P<0.05. (**j**) Survival curves of MM patients with low expression versus high expression of CIP2A (*P*=0.033).

First, we detected the characterization of MM cell lines. U266, RPMI 8226 (8226), MM.1S and MM.1R were exposed to various concentrations of Dex (2.5–80 μM) for 24 h. The half-maximal inhibitory concentration of Dex against MM.1S and 8226 cells is 0.76 and 4.53 μM, whereas half-maximal inhibitory concentration of Dex against MM.1R and U266 cells is 3.78 and 13.42 μM. As shown in Figures 1a and b, the Dex cytotoxicity was higher in MM.1S and 8226 cells than in MM.1R and U266 cells. Next, CIP2A expression was studied by QPCR in MM cell lines U266, 8226, MM.1S and MM.1R. Previous studies reported that CIP2A is overexpressed in AML and associated with human leukemia cell line HL-60 cells proliferation and differentiation.^14,24^ Thus, we used HL-60 as a positive control to test CIP2A expression in MM. The results showed that *CIP2A* is overexpressed in MM cell lines at the messenger RNA level. Interestingly, *CIP2A* expression levels in Dex-resistant MM.1R and U266 cells were higher than those in Dex-sensitive MM.1S and 8226 cells (Figure 1c). In addition, CIP2A protein levels were detected by western blot. The results show that CIP2A protein expression is also higher and consistent with the messenger RNA expression levels in Dex-resistant MM.1R and U266 cells (Figure 1d). A previous study reported that the traditional chemotherapeutic agent doxorubicin downregulates CIP2A expression, and that increased CIP2A expression confers doxorubicin resistance in breast cancer cells.^16^ Altogether, these data indicate that *CIP2A* is overexpressed in MM cell lines at the protein and messenger RNA levels. The higher CIP2A expression in Dex-resistant cells is worthy of further investigation.

As the MM cell lines presented higher CIP2A expression, we next sought to investigate whether patients with MM also express high levels of CIP2A. *CIP2A* expression was investigated by QPCR in 10 patients’ CD138^+^ cells (Figure 1e) and 4 healthy donors’ peripheral blood mononuclear cells (Figure 1f). HL-60 cell line was also used as a positive control. The results indicate that *CIP2A* was expressed at relatively high levels in all patients, but expressed at relatively low levels in normal peripheral blood mononuclear cells from four healthy volunteers. Furthermore, we determined CIP2A expression in clinical samples using immunohistochemistry analysis in 41 MM specimens and 22 adjacent normal bone trabecula and BM tissues, and determined that CIP2A was overexpressed in 46.3% of the tumor samples (19 of 41), whereas most of the adjacent normal tissues exhibited undetectable or low CIP2A staining (Figures 1g and h). In 16 cases MM tissues with paired adjacent non-tumor tissues, we observed a significantly higher expression of CIP2A in tumor tissues compared with paired adjacent non-tumor tissues (*P*<0.05, Figure 1i). These results indicated that CIP2A might be a critical molecule in MM development.

Furthermore, we analyzed the relationship between CIP2A expression levels and clinicopathological characteristics. As shown in Table 1, no significant correlation was observed between CIP2A expression and age, gender or type of myeloma (*P*>0.05). However, both International Staging System stage (*P*>0.05) and percent of BM plasma cells (*P*=0.045) show significant correlations with high level of CIP2A expression. Survival analysis revealed that MM patients with high CIP2A expression (*n*=19) presented with a poorer overall survival than those with low CIP2A expression (*n*=22; *P*=0.033; Figure 1j) And, the median survival of low and high CIP2A groups were 66 and 45 months, respectively. Altogether, our data suggested that CIP2A is overexpressed in MM, and high levels of CIP2A expression are a prognostic predictor of progression and poor prognosis of patients with MM.

### Effect of CIP2A expression on MM cell proliferation

**Figure 2:**
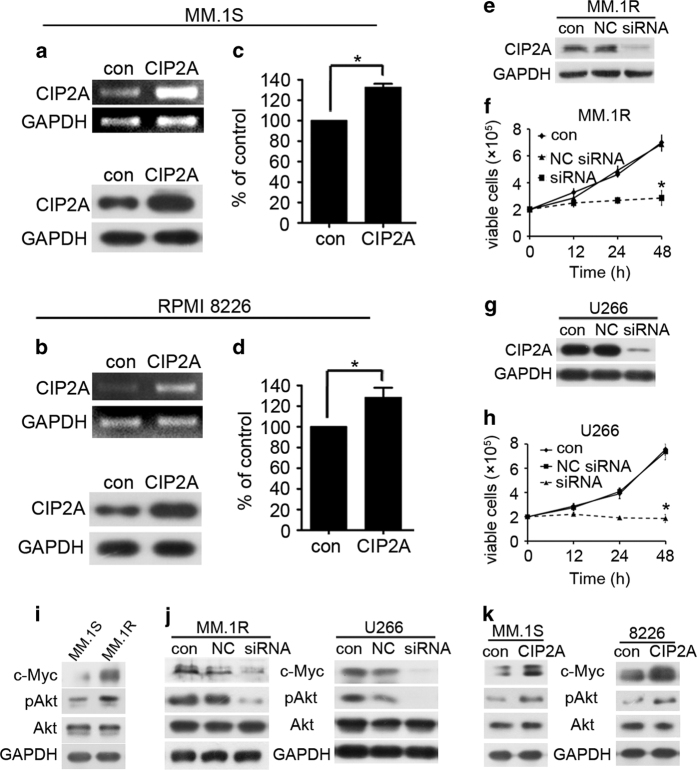
Effect of CIP2A expression on proliferation of MM cells. (**a**) MM.1S cells were transfected with a CIP2A expression plasmid, and total RNA was isolated 24 h after transfection and then subjected to reverse transcription PCR (RT-PCR) analysis, total protein was isolated and then subjected to western blot analysis. (**b**) 8226 cells were transfected with a CIP2A expression plasmid, and total RNA was isolated 24 h after transfection and then subjected to RT-PCR analysis, total protein was isolated and then subjected to western blot analysis. (**c**) MM.1S cells were transfected with a CIP2A expression plasmid, and then MTT was used to detect proliferation 24 h after transfection. (**d**) 8226 cells were transfected with a CIP2A expression plasmid, and then MTT was used to detect proliferation 24 h after transfection. (**e**) MM.1R cells were transfected with a CIP2A siRNA, and total protein was isolated 48 h after transfection and then subjected to western blot analysis. (**f**) MM.1R cells were transfected with a CIP2A siRNA, and then the number of viable cells was counted using a hemoatocytometer 48 h after transfection. (**g**) U266 cells were transfected with a CIP2A siRNA, and total protein was isolated 48 h after transfection and then subjected to western blot analysis. (**h**) U266 cells were transfected with a CIP2A siRNA, and then the number of viable cells was counted using a hemoatocytometer 48 h after transfection. (**i**) MM.1S or MM.1R cells were lysed and then subjected to western blot analysis. (**j**) MM.1R or U266 cells were transfected with a CIP2A siRNA, and total protein was isolated 48 h after transfection and then subjected to western blot analysis. (**k**) MM.1S or 8226 cells were transfected with CIP2A expression plasmid, and total protein was isolated 48 h after transfection and then subjected to western blot analysis. *P<0.05.

To identify whether an increase in CIP2A expression was associated with MM cell proliferation, MM.1S and 8226 cells were first transfected with CIP2A plasmid, and CIP2A expression was confirmed by using reverse transcription PCR and western blot (Figures 2a and b). CIP2A was highly expressed at both the messenger RNA and protein levels. CIP2A overexpression resulted in a significant increase in the proliferation of MM.1S and 8266 cells (Figures 2c and d, *P*<0.05). To confirm this result, MM.1R and U266 cells were transfected with CIP2A siRNA, and CIP2A expression was examined by using western blot analysis (Figures 2e and g). CIP2A level was markedly decreased by the CIP2A siRNA treatment, and CIP2A knockdown resulted in a significant decrease in proliferation (*P*<0.05, Figures 2f and h). The expression level of CIP2A–PP2A axis substrates (c-Myc and Akt)^5,25^ were investigated in MM.1S and MM.1R cells. The results showed that c-Myc and phosphorylated-Akt (pAkt) were more overexpressed in Dex-resistant MM.1R cells than that in MM.1S cells (Figure 2i). Furthermore, we found that CIP2A silencing resulted in the downregulation of c-Myc and pAkt (Figure 2j). And, CIP2A overexpression resulted in the upregulation of c-Myc and pAkt (Figure 2k). The c-Myc oncoprotein and Akt are capable of conferring a selective advantage to cancer cells by stimulating proliferation, cell survival, metastasis and drug resistance.^26,27^ Thus, CIP2A promotes MM cell proliferation, at least in part, through Akt and c-Myc activation.

### Effect of CIP2A expression on the Dex-mediated inhibition of MM cell proliferation

**Figure 3:**
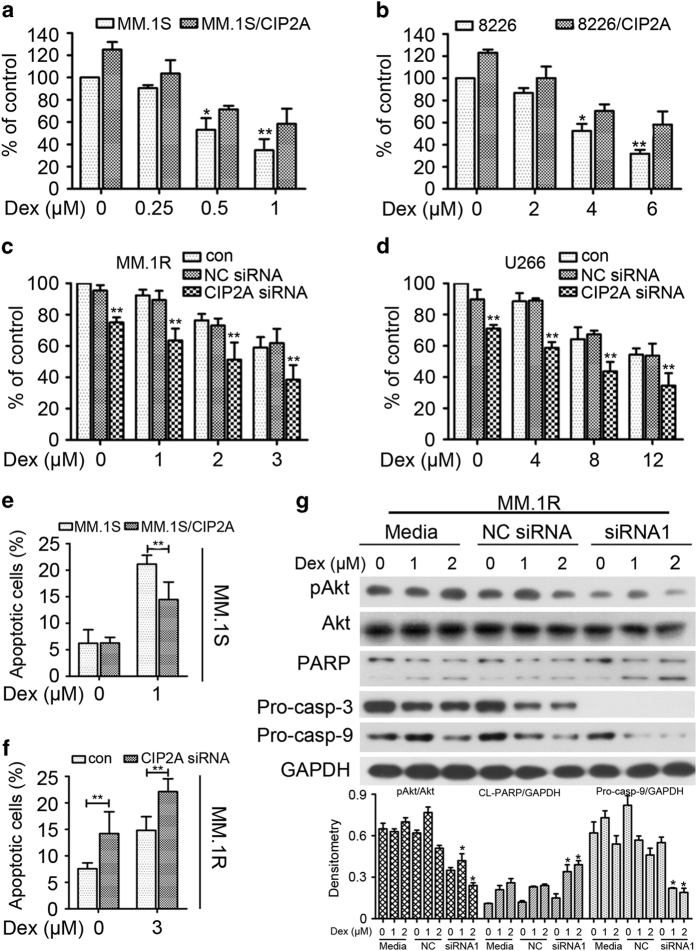
Effect of CIP2A expression on the Dex-mediated inhibition of MM cell proliferation. (**a**, **b**) MM.1S or 8226 cells were transfected with or without CIP2A expression plasmid. Twenty-four hours after transfection, MTT was used to detect the cytotoxicity of Dex. (**c**, **d**) MM.1R or U266 cells were transfected with 100 nM CIP2A or negative control (NC) siRNA. And, 48 hours after transfection the cells were then subjected to MTT to detect the cytotoxicity of Dex. (**e**) MM.1S cells were transfected with or without CIP2A expression plasmid. Twenty-four hours after transfection, cells were treated with Dex for 24 h then subjected to flow cytometry to detect apoptosis. (**f**) MM.1R cells were transfected with 100 nM CIP2A or NC siRNA. And forty-eight hours after transfection the cells were treated with Dex for 24 h and then subjected to flow cytometry to detect apoptosis. (**g**) MM.1R cells were transfected with 100 nM CIP2A or NC siRNA, followed by treatment with Dex (0, 1 and 2 μM) for 24 h. Western blot was performed using antibodies indicated. *P<0.05, **P<0.01.

To determine whether the difference in sensitivity to Dex was caused by the difference in CIP2A expression levels, MM.1S and 8226 cells were transiently transfected with CIP2A expression plasmid and then treated with Dex. CIP2A overexpression overcame the Dex-mediated inhibition of cell proliferation (Figures 3a and b). To confirm this result, MM.1R and U266 cells were transfected with CIP2A siRNA before Dex treatment. The reduced expression of CIP2A enhanced the Dex-mediated inhibition of cell proliferation (Figures 3c and d). Furthermore, flow cytometry was used to detect apoptosis. The results suggested that CIP2A overexpression overcame the Dex-induced MM.1S cell apoptosis (*P*<0.01, Figure 3e). In addition, CIP2A depletion enhanced the Dex-induced MM.1R cell apoptosis (*P*<0.01, Figure 3f). To gain insights into the molecular mechanisms underlying the enhancing effect of reduced CIP2A expression on Dex effects, MM.1R cells were transfected with CIP2A siRNA, followed by Dex treatment. Western blot analysis was used to detect variations in the protein expression (Figure 3g). Interestingly, CIP2A silencing enhanced Dex-induced apoptosis. These results imply that CIP2A expression levels determine the sensitivity of MM cells to Dex.

### Inhibition of PP2A is essential for CIP2A-induced proliferation and Dex therapy

**Figure 4:**
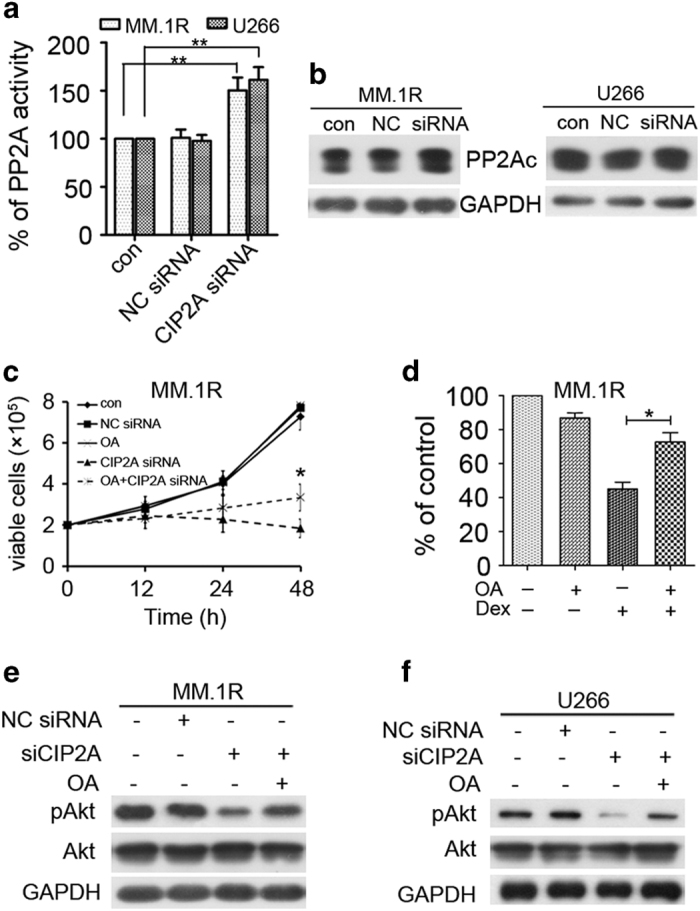
Inhibition of PP2A is essential for CIP2A-induced proliferation and Dex therapy. (**a**) MM.1R or U266 cells were transfected with a CIP2A siRNA, and PP2A activity was measured by PP2A phosphatase assay 48 h after transfection. (**b**) MM.1R or U266 cells were transfected with 100 nM CIP2A or negative control (NC) siRNA, and total protein was isolated 48 h after transfection and then subjected to western blot analysis. (**c**) MM.1R cells were transfected with CIP2A siRNA alone or in combination with 10 nM okadaic acid (OA) for 12, 24 and 48 h; cell viability was evaluated using a hemoatocytometer. (**d**) MM.1R cells were transfected with 100 nM CIP2A. And, 48 h after transfection, the cells were treated with Dex (3 μM) and/or OA (10 nM), and then subjected to CCK-8 to detect the proliferation. (**e**, **f**) MM.1R or U266 cells were transfected with CIP2A siRNA alone or in combination with 10 nM OA for 48 h. Whole-cell extracts were prepared and examined by western blot using indicated antibodies. *P<0.05, **P<0.01.

CIP2A participates in the regulation of molecular processes mostly by inhibiting the tumor suppressor PP2A.^5^ Thus, we analyzed whether CIP2A deregulation can alter the effects of the tumor suppressor PP2A in MM cells. As expected, PP2A activation was observed after CIP2A silencing (*P*<0.01, Figure 4a). Western blot analysis indicated that CIP2A silencing had no effect on PP2Ac (catalytic subunit) expression, but upregulated PP2A activity (Figure 4b). To evaluate whether CIP2A-induced cell proliferation results from the inhibition of PP2A activity, we compared cell proliferation in CIP2A siRNA-expressing MM.1R cells and MM.1R cells in the presence and absence of the PP2A inhibitor, okadaic acid (OA). Our results showed that OA treatment significantly abrogated the effect of CIP2A silencing on proliferation (*P*<0.05, Figure 4c). Moreover, the sensitivity of CIP2A-silenced MM.1R cells to Dex was also markedly inhibited by OA (*P*<0.05, Figure 4d). We next examined whether PP2A inhibition had any effects on the phosphorylation status of Akt, a target of PP2A. As expected, CIP2A knockdown decreased the phosphorylation of Akt without affecting its expression levels. Moreover, OA treatment rescued Akt phosphorylation in CIP2A knockdown U266 cells, as well as in CIP2A knockdown MM.1R cells (Figures 4e and f). Taken together, these findings suggested that PP2A inhibition is essential for CIP2A-induced proliferation and Dex therapy.

### CIP2A promotes tumorigenesis *in vivo*

**Figure 5:**
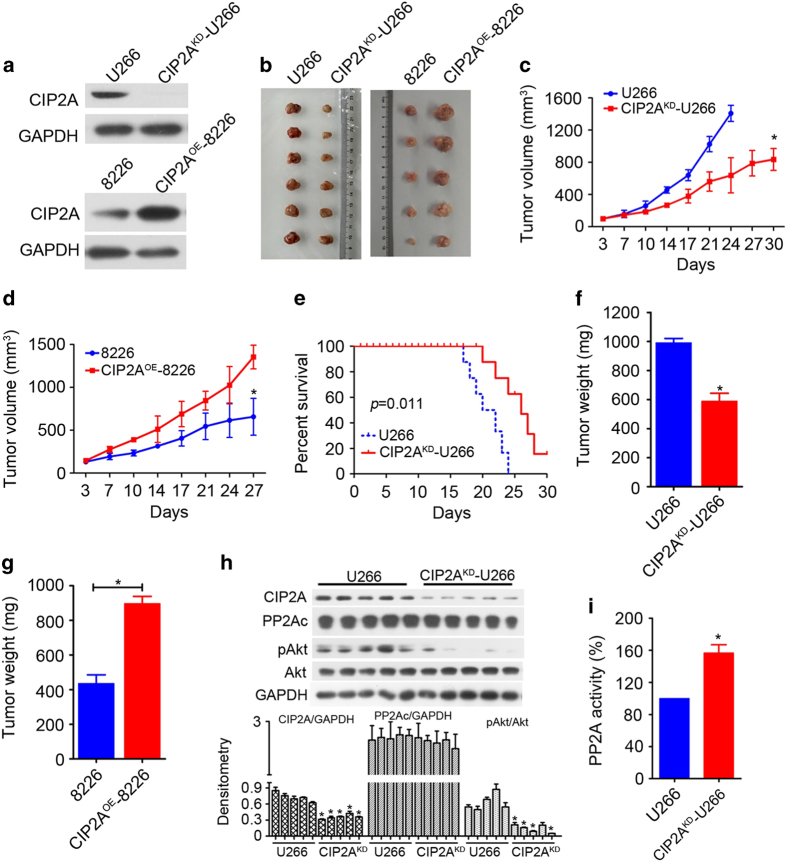
CIP2A promotes tumorigenesis *in vivo.* (**a**) Western blot of CIP2A from U266 cell lines, CIP2A^KD^-U266, 8226 and CIP2A^OE^-8226 cell lines selected with puromycin for 3 weeks after transfection. (**b**) Images of xenograft tumors obtained from mice. (**c**) CIP2A knockdown significantly inhibited MM tumor growth (CIP2A^KD^-U266 group versus U266 group). (**d**) CIP2A overexpression significantly promoted MM tumor growth (CIP2A^OE^-8226 group versus 8226 group). (**e**) Survival curve of CIP2A^KD^-U266 and U266 group mice. (**f**, **g**) Weight of the tumor from each group taken out from the mice being killed at the end of the study. (**h**) Tumor tissues were collected from mice; whole-tissue lysates were subjected to western blot using indicated antibodies. (**i**) Tumor tissues were collected from mice, and PP2A activity was measured by PP2A phosphatase assay. *P<0.05, **P<0.01.

Because of the poor tumorigenicity of MM.1R and MM.1S cells, U266 and 8226 cells were used to establish the MM murine model to test the efficacy of CIP2A.^22^ Two cell lines, CIP2A knockdown U266 (designed CIP2A^KD^-U266) and CIP2A overexpression 8226 (designed CIP2A^OE^-8226), were established respectively to further address the functional role of CIP2A in MM tumorigenesis *in vivo*. After 3 weeks of selection following Lipofectamine 3000 transfection, the CIP2A protein levels were confirmed by western blot (Figure 5a). Four groups of nude mice were subcutaneously inoculated into the right flank with U266, CIP2A^KD^-U266 cells, 8226 or CIP2A^OE^-8226, respectively. Animals were killed when the tumors reached 1.5 cm in diameter or when paralysis or major issues compromising in their quality of life occurred. After 1 month, the mice were killed and tumor tissues were isolated. Intriguingly, CIP2A^KD^-U266-transplanted mice presented fewer and smaller detectable tumor nodules compared to U266 transplanted mice (*P*<0.05, Figures 5b and c). CIP2A^OE^-8226-transplanted mice presented more and larger detectable tumor nodules compared to 8226-transplanted mice (*P*<0.05, Figure 5d). Consistent with these data, the survival of CIP2A^KD^-U266-transplanted mice was prolonged compared to that of U266-transplanted mice (Figure 5e). In addition, CIP2A depletion significantly reduced the tumor weight (*P*<0.05, Figure 5f), and CIP2A overexpression significantly increased the tumor weight (*P*<0.05, Figure 5g). Tumors from mice bearing U266 or CIP2A^KD^-U266 cells were isolated, cells were collected and experiments were conducted to determine whether CIP2A silencing influenced its downstream molecules. As shown in Figure 5h, the level of Akt phosphorylation in CIP2A^KD^-U266 tumors was significantly decreased compared to that in U266 tumors. Meanwhile, consistent with the above-mentioned results, PP2A activity was significantly increased in CIP2A^KD^-U266 tumors (*P*<0.05, Figure 5i). Altogether, these results indicated that blocking CIP2A/PP2A signaling could inhibit cell proliferation in MM.

## Discussion

In this study, the evidence supporting that CIP2A is associated with Dex therapy and poor prognosis in MM was proposed at the very first time. Previous reports indicated that the *CIP2A* gene is expressed at high levels in cancer cells from the B lymphocyte and myeloid lineages.^14,28,29^ Li *et al.*^29^ used immunohistochemistry and QPCR to confirm that CIP2A is overexpressed in BM cells from patients with a group of high-risk myelodysplastic syndromes and might play a role in the proliferation of blasts in the myelodysplastic syndromes BM and in disease progression in at least some cases. In addition, Lilja *et al.*^30^ used immunohistochemistry to show that increased CIP2A expression in B-cell lymphoma cells is correlated with increased aggressiveness of the lymphoma subtypes. However, there are fewer reports about CIP2A expression in plasma cancer cells and its function in plasma cancer. Wang *et al.*^14^ reported that CIP2A is overexpressed in AML and associated with HL-60 cell proliferation and differentiation. Thus, we used CIP2A expression levels in HL-60 as a standard and demonstrated that CIP2A is expressed in four MM cell lines, U266, 8226, MM.1S and MM.1R (Figures 1c and d). Interestingly, CIP2A expression levels were higher in Dex-resistant MM.1R and U266 cells than in Dex-sensitive MM.1S and 8226 cells, suggesting that CIP2A may play a critical role in Dex resistance.

Increasing evidence demonstrates that higher CIP2A expression promotes cancer progression, suggesting that CIP2A could be an attractive therapeutic target for the treatment of human cancers.^1,31^ Next, we investigated the CIP2A expression status and its correlation with clinicopathological features of patients with MM. Our data showed that CIP2A expression was increased in MM patients’ cells compared with that in adjacent normal bone trabecula and BM tissues (Figures 1e–i). We also demonstrated that higher CIP2A staining was significantly correlated with International Staging System stages, percent of BM plasma cells and worse survival in patients with MM (Figure 1j; Table 1). These findings indicate that CIP2A may be involved in the progression of MM and could be a significant prognostic factor for patients with MM. Although more clinical trials are required to validate this finding, we hope that CIP2A can be used as a biomarker to improve the prognostic accuracy for patients with MM.

The clinical results urged us to carry out a series of *in vitro* and *in vivo* experiments to explore the potential mechanisms underlying the effects of CIP2A. In several types of solid cancer cells, CIP2A depletion leads to impaired proliferation, foci formation, cell migration, invasion and doxorubicin resistance.^6,16,32^ Similarly, our results showed that CIP2A overexpression significantly increased cell proliferation (Figures 2a–d). RNA interference experiments showed that CIP2A silencing leads to a decrease in the proliferation of MM cells, indicating that CIP2A deregulation is an alteration that plays a potential oncogenic role in MM (Figures 2e–h). In addition, CIP2A depletion significantly inhibited the expression of c-Myc and pAkt, two critical substrates of the CIP2A-PP2A axis (Figure 2j). c-Myc is a cellular proto-oncogene associated with a variety of human cancers and is strongly implicated in the control of cellular proliferation.^10^ Given the critical role of c-Myc in promoting cell proliferation, CIP2A-mediated c-Myc stabilization may be required for sustained proliferation of MM cells. Our data demonstrated that CIP2A depletion significantly decreased c-Myc expression. Loss of c-Myc is associated with cell cycle transition-controlled proliferation.^33^ Thus, our results showed that decreased level of CIP2A correlated with c-Myc, which regulates the proliferation of tumor cells, suggesting that CIP2A plays an important role in the control of MM cell proliferation.

In addition, CIP2A was expressed at higher levels in Dex-resistant MM.1R cells, which indicated that CIP2A may participate in Dex therapy. Therefore, we tested the function of CIP2A in Dex therapy. The results showed that the increase of CIP2A expression is associated with Dex resistance and the decrease of CIP2A expression is associated with Dex sensitivity (Figures 3a–d). Moreover, CIP2A depletion promoted the effects of Dex-induced apoptosis (Figures 3e and f). Previous studies reported that Akt is a desirable target in MM, a locus and fragility of cancer multidrug resistance.^27,34^ We further examined whether the expression of pAkt was altered in the CIP2A-depleted MM.1R cells. As shown in Figure 3g, CIP2A depletion leads to pAkt downregulation and the promotion of Dex-induced apoptosis. Previous studies reported that Akt drives glucocorticoid resistance in acute lymphoblastic leukemia.^13^ These results suggest that CIP2A may contribute to Dex resistance through the inhibition of CIP2A-Akt activation.

CIP2A is a cellular PP2A inhibitor, and the CIP2A–PP2A axis is important for CIP2A-induced tumorigenesis.^1^ PP2A is a key cellular serine–threonine phosphatase and is an essential tumor suppressor.^35^ Our finding that CIP2A is frequently overexpressed in MM is novel and suggests CIP2A deregulation as a possible contributing mechanism to inhibit PP2A. Overexpression of PP2A inhibitors may play an important role in the development of human MM. Therefore, CIP2A inhibition could be a viable strategy to post-translationally target PP2A and inhibit tumor growth in MM. Indeed, CIP2A knockdown significantly increased PP2A activity (Figures 4a and b) and decreased the level of pAkt, as well as the expression of c-Myc (Figure 2j). More importantly, inhibition of PP2A activity reversed these effects (Figures 4c–f). Hence, PP2A inhibition is essential for CIP2A-induced proliferation.

*In vivo*, CIP2A depletion in MM cells significantly inhibited the formation of tumors and CIP2A overexpression significantly promoted the formation of tumors in nude mice (Figures 5b–d, f and g). Moreover, trends of c-Myc, pAkt and PP2Ac expression, and the PP2A activity of CIP2A^KD^-U266- and U266-transplanted mice were consistent with the results of our *in vitro* experiments (Figures 5h and i). CIP2A promoted MM proliferation by inhibiting PP2A activity and promoting c-Myc and pAkt expression.

In conclusion, in the present study, we demonstrated that CIP2A is overexpressed and plays an oncogenic role in MM. CIP2A inhibition may provide an important therapeutic strategy for the treatment of MM and Dex-resistant patients by targeting PP2A and facilitating the downregulation of several PP2A-regulated targets, including Akt and c-Myc. On the basis of the data presented here, CIP2A might serve as a molecular target for the development of future MM therapeutics.

## Figures and Tables

**Table 1:** Characteristics of CIP2A expression in MM patients

*Characteristics*	*Cases*	*CIP2A expression*			P-*value*^a^
	*All (*n*=41)*	*Low (*n*=22)*	*High (*n*=19)*		
	*No.*	*No.*	*No.*	*%*	
*Age (years)*					
<60	25	13	12	48	0.790
⩾60	16	9	7	43.8	
					
*Gender*					
Male	22	12	10	45.5	0.903
Female	19	10	9	47.4	
					
*Type of myeloma*					
IgG	21	11	10	47.6	0.96
IgA	9	5	4	44.4	
IgD	0	0	0	0	
IgM	1	1	0	0	
Light chain	10	6	4	40	
Nonsecretory	0	0	0	0	
					
*ISS stage*					
I	8	5	3	37.5	<0.01
II	11	7	4	36.4	
III	22	10	12	54.5	
					
*BM plasma cells (%)*					
<30	22	15	7	31.8	0.045
⩾30	19	7	12	63.2	
					
*Platelet (10*^*9 *^*l*^*−1*^)					
<130	18	7	11	61.1	0.093
⩾130	23	15	8	34.8	
					
*Hb (g l*^*−1*^)					
<100	18	8	10	55.6	0.295
⩾100	23	14	9	39.1	
					
*Albumin (g l*^*−1*^)					
<30	20	9	11	55	0.278
⩾30	21	13	8	38.1	
					
*β*_*2*_*-M (mg l*^*−1*^)					
<3.5	24	12	12	50	0.577
⩾3.5	17	10	7	41.2	

Abbrebiations: BM, bone marrow; ISS, International Staging System; MM, multiple myeloma.

CIP2A immunopositivity was graded in 1–3 scores for each patient based on the intensity of the immunoreactivity in the cancer cells, that is, 3 (+++) was strong (positive cells⩾60%), 2 (++) moderate (30%⩽positive cells<60%), 1 weak (+) (10%⩽positive cells<30%) and 0 negative (positive cells<10%).

a *χ*^2^ test.
