# Correction: Overexpression of CIP2A is associated with poor prognosis in multiple myeloma

**DOI:** 10.1038/s41392-025-02225-8

**Published:** 2025-04-12

**Authors:** Xuewen Liu, Wei Cao, Shanshan Qin, Te Zhang, Junnian Zheng, Ying Dong, Pinghong Ming, Qian Cheng, Zheng Lu, Yang Guo, Baofu Zhang, Ying Liu

**Affiliations:** 1 Laboratory of Molecular Target Therapy of Cancer, Institute of Basic Medical Sciences, Hubei University of Medicine, Shiyan, China; 2 MOE Key Laboratory of Industrial Fermentation Microbiology, College of Biotechnology, Tianjin University of Science and Technology, Tianjin, China; 3 Jiangsu Center for the Collaboration and Innovation of Cancer Biotherapy, Cancer Institute, Xuzhou Medical University, Xuzhou, China; 4 Department of Oncology, The Second Affiliated Hospital, College of Medicine, Zhejiang University, Hangzhou, China; 5 Department of Pathology, Zhuhai Hospital Affiliated with Jinan University, Zhuhai, China

Correction to: *Signal Transduction and Targeted Therapy* 10.1038/sigtrans.2017.13, published online 26 May 2017

After publication of the article,^1^ the author noticed inadvertent errors in the GAPDH Western blot panels of MM.1R cells (control, NC siRNA, and siCIP2A) in Figure 2e, as well as the GAPDH Western blot panels of MM.1R cells (control, NC siRNA, siCIP2A, siCIP2A+OA) in Figure 4e. A naming error during the scanning and storage of the film led to the GAPDH from other groups being mistakenly saved as the corresponding GAPDH for Figure 2e and Figure 4e, causing the incorrect GAPDH to be inadvertently used in these figures. In addition, the author noticed that the GAPDH Western blot panels of U266 cells (control, NC siRNA, and siCIP2A) in Figure 2g are identical to those in Figure 2j. Since these represent analyses of the same cell samples from identical experimental groups, this does not constitute an error. Nevertheless, for the sake of rigor, we decided to re-present the GAPDH in Figure 2g. The conclusion of the original article or the context of the article was not affected. The authors apologize for any inconvenience caused to the journal and readers. The incorrect part figures and corrected figures and legends are presented as follows.

Incorrect section of Figure 2e and the section of Figure 2g requiring replacement
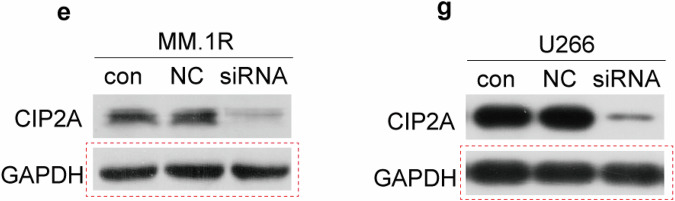


Incorrect section of Figure 4e
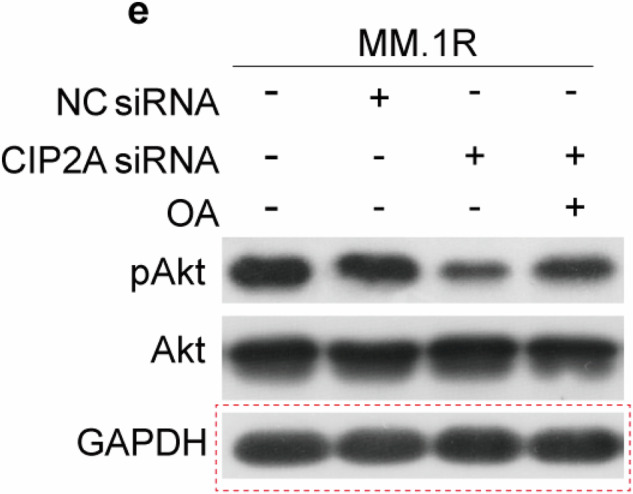


Correct Figure 2
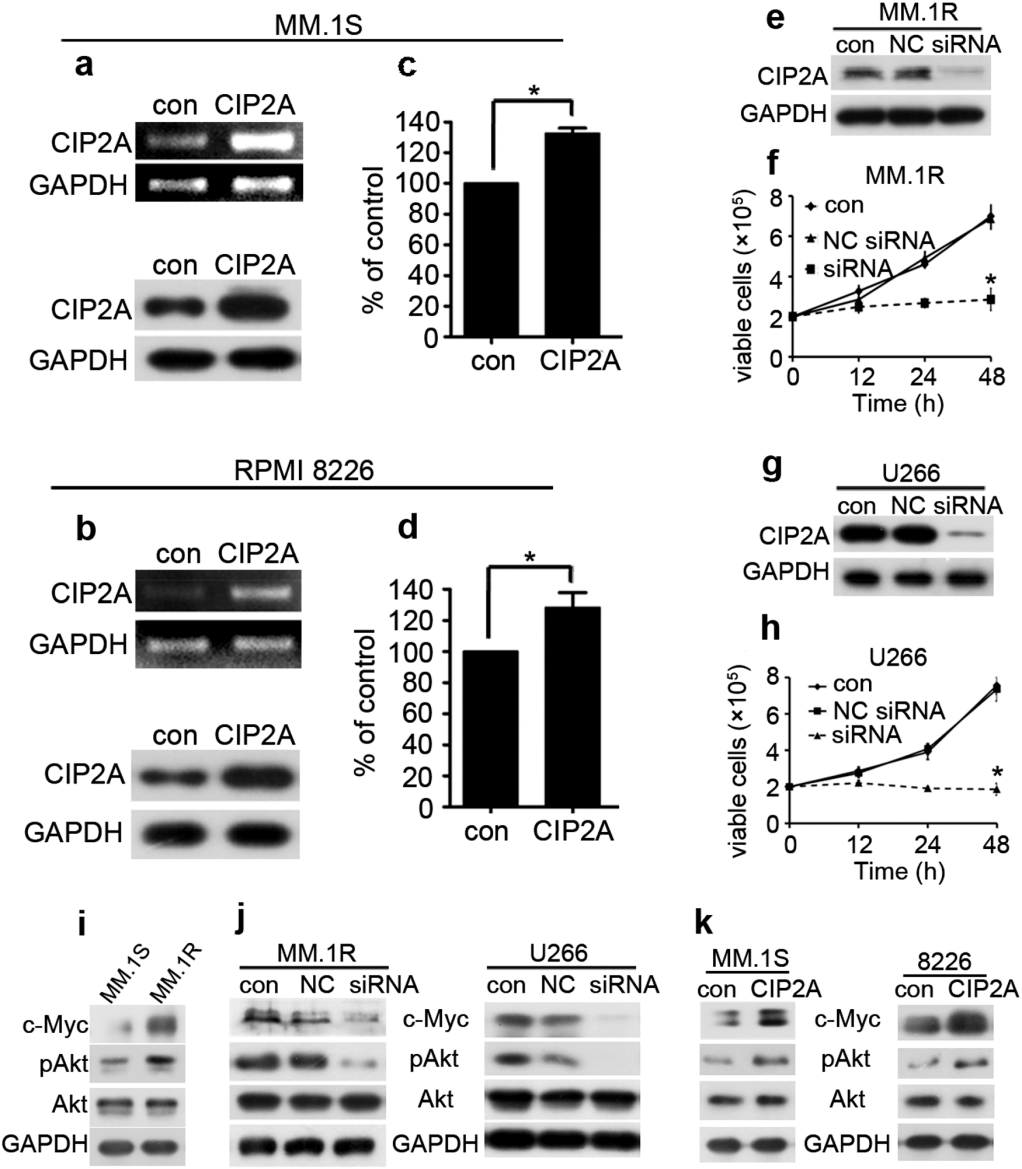


**Fig 2**. Effect of CIP2A expression on proliferation of MM cells. (**a**) MM.1S cells were transfected with a CIP2A expression plasmid, and total RNA was isolated 24 h after transfection and then subjected to reverse transcription PCR (RT-PCR) analysis, total protein was isolated and then subjected to western blot analysis. (**b**) 8226 cells were transfected with a CIP2A expression plasmid, and total RNA was isolated 24 h after transfection and then subjected to RT-PCR analysis, total protein was isolated and then subjected to western blot analysis. (**c**) MM.1S cells were transfected with a CIP2A expression plasmid, and then MTT was used to detect proliferation 24 h after transfection. (**d**) 8226 cells were transfected with a CIP2A expression plasmid, and then MTT was used to detect proliferation 24 h after transfection. (**e**) MM.1R cells were transfected with a CIP2A siRNA, and total protein was isolated 48 h after transfection and then subjected to western blot analysis. (**f**) MM.1R cells were transfected with a CIP2A siRNA, and then the number of viable cells was counted using a hemoatocytometer 48 h after transfection. (**g**) U266 cells were transfected with a CIP2A siRNA, and total protein was isolated 48 h after transfection and then subjected to western blot analysis. (**h**) U266 cells were transfected with a CIP2A siRNA, and then the number of viable cells was counted using a hemoatocytometer 48 h after transfection. (**i**) MM.1S or MM.1R cells were lysed and then subjected to western blot analysis. (**j**) MM.1R or U266 cells were transfected with a CIP2A siRNA, and total protein was isolated 48 h after transfection and then subjected to western blot analysis. (**k**) MM.1S or 8226 cells were transfected with CIP2A expression plasmid, and total protein was isolated 48 h after transfection and then subjected to western blot analysis. *P < 0.05.

Correct Figure 4
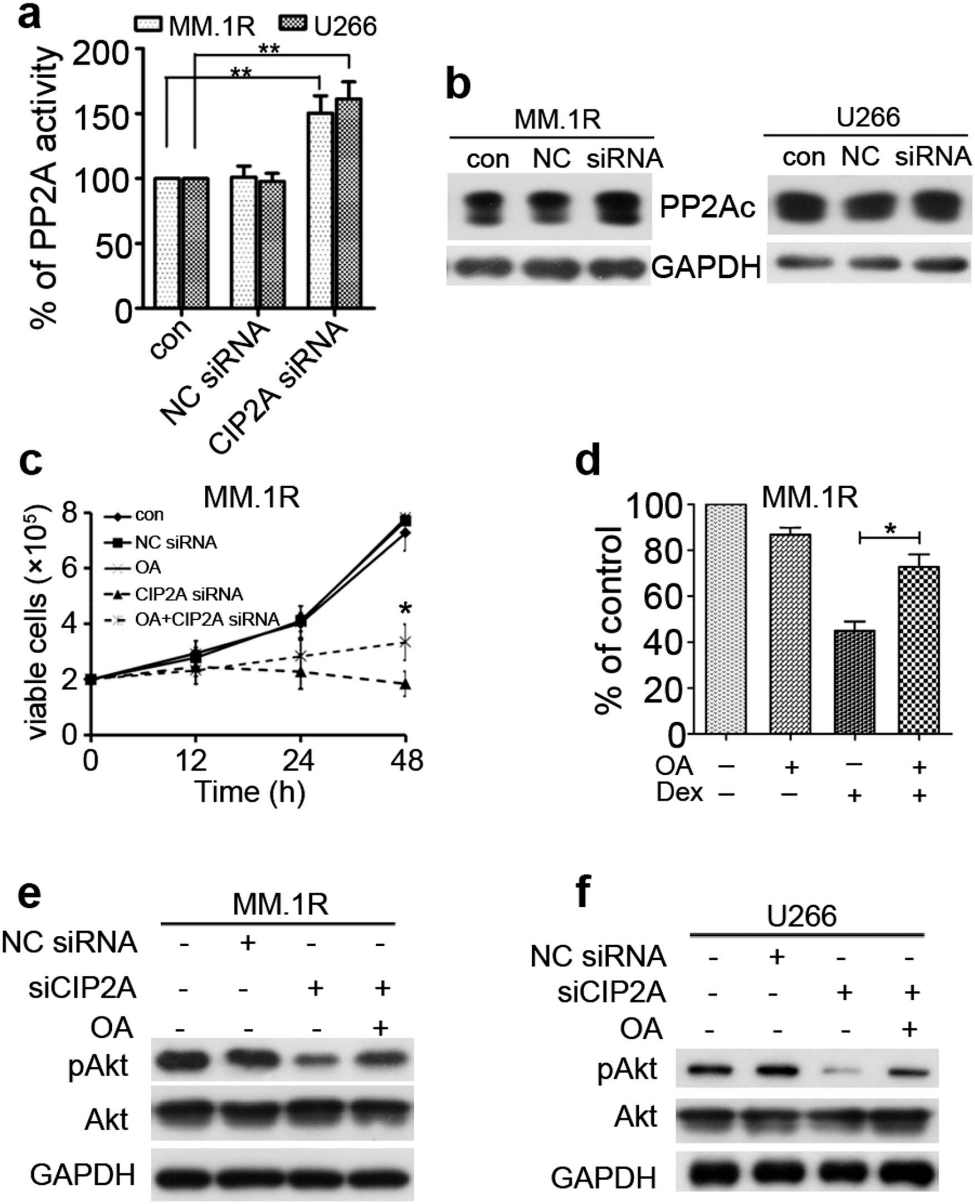


**Fig 4**. Inhibition of PP2A is essential for CIP2A-induced proliferation and Dex therapy. (**a**) MM.1R or U266 cells were transfected with a CIP2A siRNA, and PP2A activity was measured by PP2A phosphatase assay 48 h after transfection. (**b**) MM.1R or U266 cells were transfected with 100 nM CIP2A or negative control (NC) siRNA, and total protein was isolated 48 h after transfection and then subjected to western blot analysis. (**c**) MM.1R cells were transfected with CIP2A siRNA alone or in combination with 10 nM okadaic acid (OA) for 12, 24 and 48 h; cell viability was evaluated using a hemoatocytometer. (**d**) MM.1R cells were transfected with 100 nM CIP2A. And, 48 h after transfection, the cells were treated with Dex (3 μM) and/or OA (10 nM), and then subjected to CCK-8 to detect the proliferation. (**e**, **f**) MM.1R or U266 cells were transfected with CIP2A siRNA alone or in combination with 10 nM OA for 48 h. Whole-cell extracts were prepared and examined by western blot using indicated antibodies. *P < 0.05, **P < 0.01.
